# Atlanta Medical College

**Published:** 1867-09

**Authors:** 


### EDITOARIAL AND MISCELLANEOUS.

### ATLANTA MEDICAL COLLEGE.

The Ninth Annual Course of Lectures in this Institution has just been concluded.

In 1855 the first course was held, and after the suspension for four years during the war, commencing at the conclusion of the session of 1861, the exercises were resumed regularly on the first of May, 1866. The duties of the term just passed, have been discharged with satisfaction to both student and teacher.

Notwithstanding the greater stringency in money matters, than at any time since the war, the class of the present year is even larger than that of last. The additional facilities offered in the practical and theoretical course, to continue through the fall and winter, and the pecuniary relief to the country from the success of agricultural operations, give assurance of a largely increased number of students in future.

There are several of the present class who remain to attend the fall and winter course, while others will return after visiting their friends at home. Doubtless, many who have not before visited the Institution, will avail themselves of the great advantages offered to the beginner, the advanced student, and the graduate. To the first, the regular examinations and lectures daily, will be of immense value in directing the mind in the proper channel of thought, and insuring his speedy and thorough acquaintance with the fundamental principles of Medicine. To those having already made some proficiency in their studies, the practical demonstrations in Medicine, Surgery, and Obstetrics, will certainly be of immense value in giving familiarity with subjects very embarrassing to the young practitioner. The manner of determining the existence and character of disease by all the general and physical signs of morbid action, will be plainly exhibited, daily, in the College Dispensory, where fifteen or more patients are prescribed for, and in the hospital of one hundred patients, situated on the College grounds.

The small sum ($15) charged for this course of eight months, makes these advantages available to many who, in these "hard times," could not otherwise obtain them. The price of board being almost as cheap as at any place in the country, very little additional expense will be incurred to that necessary in office study at home. Students of medicine, who have not graduated and those ready to commence the practice, will doubtless find it to their interest to attend, though it may not be convenient to remain through the whole course. Even two or three months thus spent, will be found to compensate even the young practitioner, who desires to acquire practical information rapidly.

This course will continue through the entire interim, between the regular sessions of the College, from the first of September to the first of the following May. Those, therefore, commencing the study of Medicine, not wishing to take a regular full course during the winter, but desire to do so next summer at this Institution, would do well to spend most of the fall and winter here.

